# Editorial: Animal models for basic and applied research in neuroscience

**DOI:** 10.3389/fvets.2023.1356032

**Published:** 2024-01-05

**Authors:** Elsayed Metwally, Tarique Hussain, Mahmoud F. Ahmed

**Affiliations:** ^1^Department of Cytology and Histology, Faculty of Veterinary Medicine, Suez Canal University, Ismailia, Egypt; ^2^Animal Sciences Division, Nuclear Institute for Agriculture and Biology College (NIAB-C), Pakistan Institute of Engineering and Applied Sciences (PIEAS), Faisalabad, Pakistan; ^3^Department of Surgery, Anesthesiology and Radiology, Faculty of Veterinary Medicine, Suez Canal University, Ismailia, Egypt

**Keywords:** neuroscience, epilepsy, neurodegenerative (Alzheimer's), animal models and general, Diagnostic Pathology

Animal models play a crucial role in advancing neuroscience research, offering insights into the intricate workings of the brain and nervous system. They provide valuable platforms for studying various neurological conditions, understanding brain functions, and testing potential treatments (1). The objective of this frontier Research Topic is to explore the recent findings that establish connections between abnormalities in nervous system functions and the onset and advancement of both acute and chronic neurodegenerative disorders such as Alzheimer's, Parkinson's, epilepsy, and more. The study of neuroscience relies on animal models to decipher the intricate workings of the human brain and nervous system. Among the various animal subjects used in research, dogs have emerged as valuable models, offering unique insights and contributions to understanding various aspects of neuroscience. Much like humans, dogs can suffer from a variety of neurological conditions, including epilepsy, Alzheimer's disease, and degenerative myelopathy. The study of these naturally occurring conditions in dogs can provide valuable data that has direct relevance to understanding and treating human neurological disorders. In addition, dogs are occasionally used as surgical models to study the effects of spinal cord and brain injuries or to test innovative neurosurgical techniques (2).

In this Research Topic of Frontiers in Veterinary Science/Veterinary Experimental and Diagnostic Pathology, five manuscripts were published: two reviews and three original research articles, whose main contributions and results are briefly presented below.

Epilepsy stands as the most prevalent neurological disorder in dogs, representing a complex brain dysfunction. This condition causes affected dogs to possess a predisposition for experiencing spontaneous epileptic seizures. The origins of epilepsy remain largely unclear, with a limited understanding of its pathogenesis. It is widely believed that a combination of various genes and environmental factors interact, ultimately leading to the manifestation of clinical symptoms (3). In this Research Topic, Rosendahl et al. revealed that dogs affected by idiopathic epilepsy exhibit elevated blood copper levels, an increased copper-to-zinc ratio, and higher selenium levels. Additionally, they demonstrate lower blood chromium levels compared to healthy dogs. These findings emphasize the significance of disturbed trace element levels in dogs diagnosed with idiopathic epilepsy. This study proved that potassium bromide treatment might influence arsenic metabolism in dogs. However, the mechanisms through which trace elements might participate in the development of canine epilepsy or seizures still require further investigation.

In another original research article published by Phochantachinda et al., it was shown that the link between epilepsy and cognitive decline has been studied in canines, revealing a common occurrence of memory impairment among dogs affected by epilepsy. The proteomic profiling they did showed that haptoglobin, ceruloplasmin, α2-macroglobulin, complement factor H, and gelsolin are all connected to epilepsy in dogs and Aβ levels. These proteins potentially serve as diagnostic biomarkers, pending clinical validation, with the potential to be utilized in veterinary practice. Moreover, they are relevant to disease response pathways.

Degenerative myelopathy is a progressive and chronic neurodegenerative spinal cord condition primarily affecting upper motor neurons. Its complexity is mainly linked to a mutation in the SOD1 gene, which plays a toxic role in the disease. Unfortunately, there is currently no specific treatment available for this condition (4). In a clinical trial by Gouveia et al., they evaluated the potential for neural regeneration in dogs with degenerative myelopathy undergoing an intensive neurorehabilitation program along with mesenchymal stem cell transplantation. The hypothesis posits that implementing this combined protocol may offer a promising avenue for a more positive and prolonged progression in these dogs. However, the study's constraints encompassed a limited sample size.

In a review article illustrated the animal sexual behavior from a neuroscience perspective, Tekin et al. showed that hormones secreted from the brain and endocrine glands regulate reproductive activity in domestic mammals. Numerous hormones play essential roles in sustaining reproductive functions.

Neurological degenerative disorders have a significant and increasing healthcare hurdle worldwide. Within the various molecular pathways involved in their development, calpain signaling has been identified as a key contributor to neuronal malfunction and the demise of cells (5). In a review article by Metwally et al., they shed light on the current functions assigned to calpains and offered an overview of the mechanisms that regulate their activity throughout the progression of neurodegenerative disorders. In addition, they discussed how calpain inhibition might be employed as a potential therapy for neuronal dysfunctions in neurodegenerative diseases such as spinal cord injury. The review emphasized an experimental model study of spinal cord injury in adult dogs that demonstrated that inhibiting calpain activity with a specific calpain inhibitor (PD150606) in combination with methylprednisolone sodium succinate resulted in decreased neuronal death, increased the canine Basso, Beattie, and Bresnahan locomotor score, and provided enhanced neuroprotection (6).

In conclusion, animal models are indispensable in neuroscience, serving as a cornerstone for understanding the intricacies of the brain and nervous system. They bridge the gap between basic understanding and practical applications, enabling groundbreaking discoveries and potential treatments for neurological conditions. As technology and ethical considerations progress, these models will continue to shape our knowledge of this vital field of medicine.

## Author contributions

EM: Writing – original draft, Writing – review & editing. TH: Writing – original draft, Writing – review & editing. MFA: Writing – original draft, Writing – review & editing.

## References

[1] Mukherjee P Roy S Ghosh D Nandi SK. Role of animal models in biomedical research: a review. Lab Anim Res. (2022) 38:18. 10.1186/s42826-022-00128-1 35778730 PMC9247923

[2] Löscher W. Dogs as a natural animal model of epilepsy. Front Vet Sci. (2022) 9:928009. 10.3389/fvets.2022.928009 35812852 PMC9257283

[3] Uriarte A Maestro Saiz I. Canine versus human epilepsy: are we up to date? J Small Anim Pract. (2016) 57:115–21. 10.1111/jsap.12437 26931499

[4] Coates JR March PA Oglesbee M Ruaux CG Olby NJ Berghaus RD. Clinical characterization of a familial degenerative myelopathy in Pembroke Welsh Corgi dogs. J Vet Intern Med. (2007) 21:1323–31. 10.1111/j.1939-1676.2007.tb01955.x 18196743

[5] Metwally E Zhao G Zhang YQ. The calcium-dependent protease calpain in neuronal remodeling and neurodegeneration. Trends Neurosci. (2021) 44:741–52. 10.1016/j.tins.2021.07.003 34417060

[6] Metwally E Al-Abbadi HA Hashem MA Mahmoud YK Ahmed EA Maaty AI. Selective calpain inhibition improves functional and histopathological outcomes in a canine spinal cord injury model. Int J Mol Sci. (2022) 23:11772. 10.3390/ijms231911772 36233068 PMC9570220

